# Complicated pylephlebitis secondary to perforated appendicitis in a child- A rare case report

**DOI:** 10.1016/j.amsu.2022.104744

**Published:** 2022-09-23

**Authors:** Soumya Pahari, Manju Shrestha, Sunil Basukala, Pooja Kafle, Kalpana Rai, Yugant Khand, Ojas Thapa, Anup Thapa

**Affiliations:** a Nepalese Army Institute of Health Sciences (NAIHS), Sanobharyang, 44600, Kathmandu, Nepal; b Children's Hospital for Eye Ent & Rehabilitation Services (CHEERS), Kathmandu, Nepal; c Department of Surgery, Shree Birendra Hospital, Chhauni, Kathmandu, 44600, Nepal; d Department of Radiology, Shree Birendra Hospital, Chhauni, Kathmandu, 44600, Nepal

**Keywords:** Pylephlebitis, Perforated appendicitis, Thrombophlebitis, Case report

## Abstract

**Introduction and importance:**

Pylephlebitis is a rare and life threatening thrombophlebitis of the portal vein. It commonly occurs following intra abdominal infections like appendicitis.

It is even rarer in the pediatric age group. The nonspecific presentation impedes the diagnosis. Timely use of appropriate antibiotics and control of infection is paramount in its treatment and this case report highlights the same.

**Case presentation:**

11 year old female child from a rural area was referred from a local hospital for persistent fever and abdominal pain despite medical treatment. Workup revealed perforated appendicitis, pylephlebitis, and multiple liver abscess. She was successfully treated with appendicectomy followed by antibiotics and anticoagulants.

**Clinical discussion:**

Pylephlebitis secondary to appendicitis was frequently lethal in the pre-antibiotic era. Doppler ultrasonography and CT scan are the investigations of choice to establish the diagnosis by showing a thrombus in the portal vein. With use of antibiotics, early diagnosis by imaging and surgical control of the primary infection, appendicitis-associated-pylephlebitis now has improved outcomes. Larger scale studies are required to establish the role of anticoagulants.

**Conclusion:**

Early diagnosis and intervention of this fatal condition is life saving but numerous gaps exist in the literature regarding the treatment recommendation.

## Highlights

• Pylephlebitis, the septic thrombophlebitis of portal vein is rare and fatal.
• It usually occurs as a complication of intra abdominal infections.
• Non specific presentation impedes it diagnosis.
• Its progression can lead to bowel ischemia, sepsis and death.
• Prompt treatment with antibiotics and control of infection is crucial for better outcome.

## 1 Introduction

Pylephlebitis is the septic thrombophlebitis of the portal vein system. It is an uncommon but life threatening complication of intra abdominal infection. It usually occurs following diverticulitis or appendicitis, and may also occur following necrotizing pancreatitis, inflammatory bowel disease, hemorrhoidal disease, perforation by foreign body, acute cholecystitis, Behcet's syndrome and amoebic colitis [1].

Nonspecific clinical presentation makes the diagnosis difficult and it may progress to mesenteric and splenic vein thrombosis causing bowel ischemia/infarction, hepatic abscess and even death [2]. The most common complication is cavernomatous transformation that may lead to portal hypertension and variceal bleeding [3].

The incidence in children is unknown as it is rare but adult studies show an incidence of 2.7 per 100000 person-year [4]. Though uncommon, undiagnosed perforated appendicitis in children may lead to pylephlebitis and only few cases have been reported. Other known conditions in children include umbilical vein catheterization, necrotizing enterocolitis, acute or chronic pancreatitis, and liver abscess [5]. Early antibiotics administration and surgical intervention has decreased the incidence to 0.05% for acute appendicitis and 3% for ruptured appendicitis [6]. The mortality rate of this life threatening condition in children have been reported to be up to 50% [4]. Diagnosis may be elusive and delayed due to non-specific presentation and requires a high index of suspicion in the background of an abdominal sepsis. Doppler ultrasonography and computed tomography (CT) scan are the investigations of choice to establish the diagnosis by showing a thrombus in the portal vein. CT has an added benefit to detect any underlying abdominal inflammatory process like appendicitis, pancreatitis, etc. Timely use of appropriate broad spectrum antibiotics and control of the septic foci are of paramount importance for better outcomes. Infection is usually polymicrobial, and the most commonly isolated organism is *Bacteroides fragilis* owing to its coagulation promoting surface and capsular components [7]. The role of anticoagulation in pylephlebitis still remains controversial.

We describe a case of a female child presenting with pylephlebitis secondary to perforated appendicitis who was successfully managed with antibiotics and anticoagulation following appendectomy. With this case report, we aim to highlight that delay in diagnosis of common conditions like acute appendicitis may be encountered especially in developing countries, leading to rarer complications like pylephlebitis in our case. Timely diagnosis and appropriate antibiotics use with control of infection is of paramount importance for better outcome in such cases.

## **2** Methods

This case is reported in line with the SCARE 2020 checklist [8].

## 3 Case presentation

An 11-year-old female child, presented at our hospital with pain in the right lower abdominal pain for the past ten days. She was from a rural part of Nepal and was in her usual state of health until four weeks before when she presented with a history of abdominal pain, low grade fever with chills accompanied by vomiting and loose stool. The abdominal pain was located in the right lower quadrant, progressive in nature and moderate in severity. She was initially treated in a local hospital near her hometown. As her pain worsened and her symptoms did not improve after repeated treatment, she visited our hospital for further medical management. Her past medical and surgical history, family history, personal history and allergic history were insignificant.

On arrival at the emergency via ambulance, she presented with persistent fever and right lower abdominal pain. She was ill-appearing, cachectic girl with a temperature of 101 °F, blood pressure of 90/60 mmHg and heart rate of 110 beats/minute. She was in tachypnea with a respiratory rate of 28 per minute. Her initial abdominal examination demonstrated tenderness in the right iliac fossa with positive rebound tenderness with normal bowel sounds but the remainder of the physical examination was unremarkable. Her laboratory values on admission showed a leukocyte count of 28,000/mm3, hematocrit of 30.5%, prothrombin time of 14.2 seconds, fibrinogen of 497 mg/dL, ALT (U/L)of 97, AST of 91 IU/L, and albumin of 2.98 g/dL (Table 1.).

**Table 1:** Laboratory parameters on admission and after surgery.

s.no	Laboratory test	Normal range	On admission	Post op
1.	WBC count (10^9^ cells/L)	3.5–9.5 10^9^ cells/L	28.0 X10^9^	11.0 X10^9^
2.	Neutrophil (%)	50–70%	90	83
3.	Lymphocyte (%)	20–40%	06	35
4.	Hemoglobin (g/dL)	11.4 12–16 g/dL	9.8 g/dL	10.3 g/dL
5.	Platelet count (109 cells/L)	125–350,10^9^	180	240
6.	PT (Prothrombin time)	11–16 secs	14.2	16
7.	Bilirubin Total mg/dL	0.4–1.2	0.5	0.6
8.	Bilirubin Conjugated	0–0.8	0.3	0.5
9.	AST (U/L)	5–45 U/L	12.5	149
10.	ALT (U/L)	5–40 U/L	97	102
11.	Albumin (g/L)	3.5–5.5 mg/L	2.98	3.3
12.	Amylase (U/L)	0–140 U/L	123	93
13.	Lipase (U/L)	0–60 U/L	48	41
14.	Blood urea nitrogen (mg/dL)	8–20 mg/dL	17.9	21
15.	Creatinine (mg/dL)	0.5–1.2 mg/dL	0.4	1.4
16.	C-Reactive Protein CRP(mg/dL)	0–6.45	305.5	123.7

**Fig. 1:**
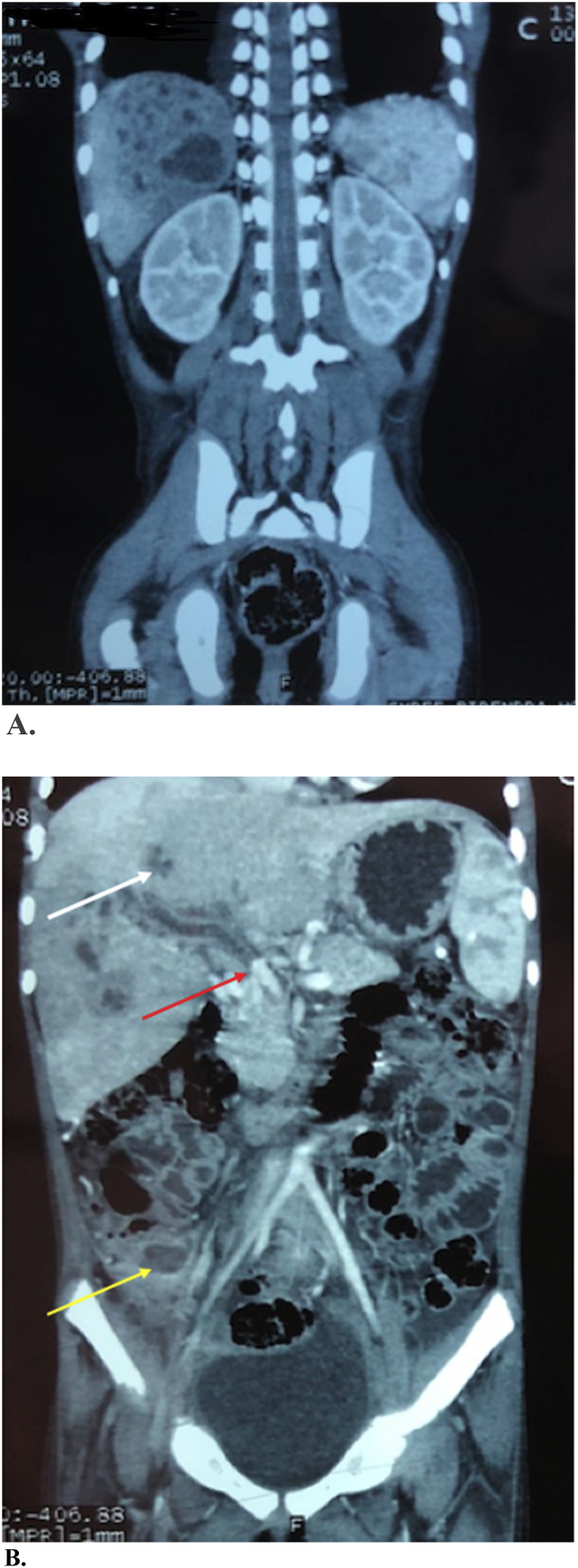
Contrast Enhanced Computed Tomography (CECT) scan of the abdomen showing A. Hepatomegaly with multiple coalescing hypodense lesion diffusely scattered in liver parenchyma with enhancing internal septations within suggestive of multiple liver abscess B. Right subcapsular hepatic abscess (White arrow), Thrombus in the main portal vein with portal vein thrombosis (Red Arrow), Appendicular abscess (Yellow arrow) with minimal fluid collection in RIF. (For interpretation of the references to colour in this figure legend, the reader is referred to the Web version of this article.)

Since the patient was hemodynamically unstable, she was admitted to the Paediatric Intensive Care Unit (PICU) for further workup. Our differential diagnosis included appendicular abscess and mesenteric lymphadenitis which were subsequently ruled out by imaging. She underwent Ultrasonography (USG) abdomen and pelvis which showed an ill-defined heteroechoic lesion with anechoic component measuring 2.7 X 3.1 × 3.3 cm in left lobe of liver with feature suggestive of hepatomegaly with liver abscess. There was a cavernous transformation of portal vein at intrahepatic part and collaterals at periportal region with intraluminal echogenic contrast suggestive of portal vein thrombosis at mid part suggestive of portal hypertension with minimal ascites. She further underwent Contrast Enhanced Computed Tomography (CECT) abdominal pelvis which confirmed hepatomegaly with multiple coalescing hypodense lesion diffusely scattered in liver parenchyma largest measuring (6.5X7.5 × 7.2 cm) in segment VII and enhancing internal septations within suggestive of lesion of infective etiology with multiple liver abscess. Portal vein thrombosis with periportal collaterals, mild ascites was also confirmed. The scan also showed wall enhancing collection in Right Iliac fossa (RIF) in close proximity to the appendix likely to be appendicular perforation with an abscess formation. (Fig. 1. A, B).

Based on the history, physical examination, and radiographic findings, a presumptive diagnosis of pylephlebitis secondary to a perforated appendix was made. She was started on broad spectrum antibiotics. An emergency open appendectomy was performed. Intraoperatively, significant abdominal ascitic fluid was noted. An appendiceal remnant in a retrocecal position with perforation on the tip of appendix was found. A periappendiceal abscess of around 10ml was found with extensive serosal fibrous adhesions consistent with perforated appendicitis. The base of the appendix was healthy and was tied and the abdomen was closed by the placement of subphrenic drain.

Postoperatively, the patient continued to have fever for the first day. On the second postoperative day, her leukocyte count significantly decreased to 11,000/mm^3^ day with resolution of abdominal symptoms. During her stay in the hospital she was prescribed an anticoagulant for portal vein thrombosis for the duration of three months. Histopathology of the appendectomy specimen confirmed perforated appendicitis. A computed tomography (CT) scan on postoperative day five showed significant reduction in the right hepatic abscesses, persistent left hepatic abscesses, and persistence without progression of the portal venous thrombosis. She then underwent ultrasound-guided percutaneous drainage of about 40 mL of pus from the left lobe of the liver. Antibiotics were continued for a total duration of 6 weeks. At 6 weeks follow up, she was asymptomatic and doing well.

## 4 Discussion

Pylephlebitis can be defined as the thrombophlebitis of the portal vein and its tributaries. Appendicitis leading to pylephlebitis in children is uncommon but a life threatening complication [4]. Other abdominal infections draining to portal vein can lead to pylephlebitis [7]. Commonly isolated organisms are Bacteroides fragilis, E coli, Proteus mirabilis, though the infection is mostly polymicrobial [7]. Nonspecific presentation makes its diagnosis difficult. The presentation may include spiking fever, right upper quadrant abdominal pain, anorexia, weight loss and malaise [6]. However, children may present with subtle findings [6]. The diagnosis is made by USG that shows thrombus in portal vein and CT (less operator dependent) [7]. It has to be promptly treated to prevent its progression to mesenteric and splenic vein that may lead to bowel ischemia, infarction, hepatic abscess and even death.

There are only a few case reports published about pylephlebitis in children secondary to perforated appendicitis.Pylephlebitis secondary to appendicitis used to be the most common cause of hepatic abscess in the pre antibiotic era (50%) and was frequently lethal [9].

In the current era of early treatment with antibiotics and timely diagnosis with imaging, incidence of pylephlebitis is rare. However, in developing countries like ours where lack of tertiary care facilities in rural areas and patient misinformation is prevalent, its incidence may be more. Diagnostic delay of pylephlebitis is attributable to its non-specific clinical presentation ranging from fever, abdominal pain, jaundice and weakness to septic shock and death [1]. Delay in diagnosis in our case can be attributable to the non specific symptoms like abdominal pain, nausea, vomiting along with lack of imaging facilities like CT scan in the rural setting where she was first treated.

An uncontrolled infection in the regions drained by the portal system initially leads to thrombophlebitis of the small mesenteric veins and eventually spread to the portal vein and liver parenchyma, leading to pylephlebitis and hepatic abscess [10]. Apart from intra abdominal sepsis, hypercoagulable states, malignancy and immunosuppressed states are additional predisposing factors. When such risk factors are present, the mortality may be more [6,11]. About 41% of pylephlebitis show associated hypercoagulable states [6], but no such comorbidities were found in our patient. The most common complication of portal vein thrombosis is cavernomatous transformation of portal veins (CTPV), which was also present in our patient. It itself has a mortality of about 10% and can lead to obstructive jaundice (portal biliopathy), portal hypertension and variceal bleeding [12]. The thrombophlebitis can extend to involve the mesenteric veins and splenic veins. Superior and inferior mesenteric thrombosis have a dreadful consequence of bowel ischemia, which has high mortality [13,14]. Despite the diagnostic delay of appendicitis, timely intervention of pylephlebitis prevented such complications in our case. With use of antibiotics, early diagnosis by imaging and surgical control of the primary infection, appendicitis-associated-pylephlebitis which was considered uniformly fatal in the past, now has improved outcomes. There are no standard guidelines or large scale studies to our knowledge that recommend the empirical regimen and duration of antibiotics treatment for pylephlebitis. Available studies recommend antibiotics duration of 4 weeks without liver abscess and 6 weeks in the setting of liver abscess with drainage if abscess size is more than 3 cm [15]. Since hepatic abscess was present and no hypercoagulable states were identified, our patient was prescribed antibiotics for 6 weeks and anticoagulants for 6 months. Percutaneous drainage of the infected portal vein has also been described as a measure to control infection [16]. There are no concrete guidelines or large scale studies regarding the role of anticoagulants in pylephlebitis and is a topic of debate. Many case reports and series published in the literature mention use of anticoagulants. It may prevent thrombus extension, improve recanalization and reduce the risk of bowel ischemia [4]. Anticoagulants may be recommended in the background of hypercoagulable states or mesenteric vein thrombosis to prevent bowel ischemia [11,17]. The clinical end point of anticoagulation is also unclear, some studies mention 3–6 months, longer duration may be required if underlying hypercoagulable state is also present [18]. Death from pylephlebitis in the modern era is attributable to onset of severe sepsis before administration of antibiotics [19].

## 5 Conclusion

Pylephlebitis secondary to appendicitis albeit rare in the era of modern medicine, may still be prevalent in developing countries. The early intervention of this fatal condition is life saving but numerous gaps exist in the literature regarding its treatment recommendation. Empirical regimen and duration of antibiotics, role of anticoagulants and its clinical endpoint if used must be clearly delineated by large scale studies. On the clinician's part, pylephlebitis must be kept in mind when the patient presents a long standing abdominal sepsis.

## Ethical approval

Not required in our case.

## Sources of funding

None.

## Author contributions

Sunil Basukala (SB) = Conceptualization, Supervision.

Sunil Basukala (SB), Soumya Pahari (SP), Manju Shrestha (MS) = Writing - original draft.

Soumya Pahari (SP), Manju Shrestha (MS), Sunil Basukala (SB), Pooja Kafle (PK), Yugant Khand (YK), Ojas Thapa (OT), Kalpana Rai (KR), Anup Thapa (AT) = Writing - review & editing.

All the authors read and approved the final manuscript.

## Registration of research studies

1. Name of the registry: Not applicable
2. Unique Identifying number or registration ID: Not applicable
3. Hyperlink to your specific registration (must be publicly accessible and will be checked): Not applicable

## Guarantor

Sunil Basukala (SB).

## Consent

Written informed consent was obtained from the patient's parent for publication of this case report and accompanying images. A copy of the written consent is available for review by the Editor-in-Chief of this journal on request.

## Provenance and peer review

Not commissioned, externally peer-reviewed.

## Declaration of competing interest

No conflict of interest.
